# Influence of image reconstruction on quantitative cardiac ^15^O-water positron emission tomography

**DOI:** 10.1007/s12350-022-03075-5

**Published:** 2022-08-04

**Authors:** Jonny Nordström, Elin Lindström, Tanja Kero, Jens Sörensen, Mark Lubberink

**Affiliations:** 1 Nuclear Medicine and PET, Department of Surgical Sciences, Uppsala University, Uppsala, Sweden; 2 Centre for Research and Development, Uppsala/Gävleborg County, Gävle, Sweden; 3 Medical Physics, Uppsala University Hospital, 75185 Uppsala, Sweden; 4 PET Center, Uppsala University Hospital, Uppsala, Sweden

## Abstract

**Background:**

The impact on quantitative ^15^O-water PET/CT of a wide range of different reconstruction settings, including regularized reconstruction by block-sequential regularized expectation maximization (BSREM), was investigated.

**Methods:**

Twenty clinical stress scans from patients referred for assessment of myocardial ischemia were included. Patients underwent a 4-min dynamic stress PET scan with ^15^O-water on a digital PET/CT scanner. Twenty-two reconstructions were generated from each scan and a clinical reconstruction was used as reference. Varied parameters were number of iterations, filter, exclusion of time-of-flight and point-spread function, and regularization parameter with BSREM. Analyses were performed in aQuant utilizing two different methods and resulting regional myocardial blood flow (MBF), perfusable tissue fraction (PTF), and transmural MBF (MBFt) values were evaluated.

**Results:**

Across the two analyses, correlations toward the reference reconstruction were strong for all parameters (ρ ≥ 0.83). Using automated analysis and the diagnostic threshold of hyperemic MBF at 2.3 mL⋅g^−1^⋅min^−1^, diagnosis was unchanged irrespective of reconstruction method in all patients except for one, where only four of the most extreme reconstruction methods resulted in a change of diagnosis.

**Conclusion:**

The low sensitivity of MBF values to reconstruction method and, as previously shown, scanner type and PET/CT misalignment, confirms that diagnostic hyperemic MBF cutoff values can be consistently used for ^15^O-water.

**Supplementary Information:**

The online version contains supplementary material available at 10.1007/s12350-022-03075-5.

## Introduction

Positron emission tomography (PET) is a valuable noninvasive tool for both diagnosis and risk stratification of coronary artery disease (CAD).^1–3^ Quantification of hyperemic myocardial blood flow (MBF) has been shown superior to qualitative analysis in the detection of significant CAD,^4–6^ and ^15^O-water PET is considered the noninvasive gold standard for quantification of MBF.^7,8^ In the clinical assessment of significant CAD, a diagnostic threshold of hyperemic MBF is utilized which has been established at 2.3 mL⋅g^−1^⋅min^−1^ for ^15^O-water.^5^ The use of a uniform absolute diagnostic threshold for pathological hyperemic MBF in clinical decision making requires robust quantification, which means, comparable values across scanners and centers, as well as reconstruction methods. Iterative image reconstruction using ordered-subsets expectation maximization (OSEM) is the most used method for clinical PET reconstruction. OSEM in combination with resolution recovery modeling (point-spread function, PSF) and time-of-flight (TOF) information has improved the image quality. Higher spatial resolution can be achieved with the inclusion of PSF modeling to the system matrix^9^ and TOF increases the rate of convergence allowing for less iterations with OSEM.^10^ Furthermore, penalized likelihood reconstruction by block-sequential regularized expectation maximization (BSREM) has in recent years become available for clinical PET reconstruction.^11^ BSREM adds regulation to the iterative process which allows for fully converged images with low noise levels and high quantitative accuracy.

Different types of reconstructions are being used across centers and this has been shown to influence both image quality and quantification. For ^82^Rb, a recent study showed a septal difference in MBF between optimal reconstruction settings including TOF compared to without TOF.^12^ On the global level, another ^82^Rb study showed an increase in MBF of about 10% for TOF + PSF compared with a standard OSEM algorithm.^13^ MBF with ^13^N-ammonia was shown to be minimally affected when comparing reconstruction by filtered back projection to OSEM (2i/24s, 6.4 mm filter) and BSREM with β-values ranging from 100 to 500.^14^ For ^15^O-water, MBF has been shown to be minimally affected by TOF, using 2 or 3 iterations, or applying a 6 or 8 mm filter with a hybrid PET/MR scanner,^15^ but this study did not include BSREM. In addition, MBF quantification from ^15^O-water has proven feasible both without attenuation correction and with an erroneously applied attenuation correction due to PET/CT misalignment.^16,17^

In the present study, the impact of a wider range of different reconstruction settings on quantitative cardiac ^15^O-water PET/CT, including BSREM algorithms, was investigated. To the best of our knowledge, this is the first study to investigate the impact of BSREM reconstruction algorithms on MBF quantification from ^15^O-water PET. In addition, impact on the perfusable tissue fraction (PTF), which can be used as a marker of viability, was also investigated which has not been done previously.

## Methods

### Patients

The study included 20 clinical stress scans from patients referred for assessment of myocardial ischemia with ^15^O-water PET/CT. All scans were acquired between September and November 2020 and the cohort consisted of six females and 14 males with mean age of 65 (range of 46-79) and mean BMI of 27 (range of 19-40). All data processing and analysis was performed on anonymized image data and the study was approved by the Swedish Ethical Review Authority (reference 2019/00092).

### PET scanning protocol

Patients were scanned using a Discovery MI PET/CT scanner (GE Healthcare, Waukesha, WI) consisting of 5 detector rings (25 cm FOV in 89 slices). For attenuation correction, a low dose CT scan during normal breathing started the protocol. Then, 4-min dynamic list mode emission stress scans were performed starting simultaneously with automated bolus injection of 400 MBq ^15^O-water (5 mL ^15^O-water at 1 mL⋅min^−1^ followed by 35 ml saline at 2 mL⋅min^−1^). A continuous infusion of adenosine (140 μg⋅kg^−1^⋅min^−1^) starting 2 minutes prior to the start of the PET acquisition and continuing throughout the whole scan was used to induce hyperemic MBF. Data were reconstructed into a dynamic series of 20 frames (1 × 10, 8 × 5, 4 × 10, 2 × 15, 3 × 20, 2 × 30 seconds) using TOF-OSEM reconstructions with different numbers of iterations (1-6i, increments of 1), filter sizes, as well as with and without TOF and PSF. BSREM reconstructions included both TOF and PSF, and β-values of 100, 200, 300, 400, 600, 800, and 1000 were used. In total, 22 different reconstructions per patient were acquired (Table 1). A matrix size of 192 × 192 with resulting voxel dimensions of 2.6 × 2.6 × 2.8 mm^3^ was used. The clinical reconstruction used at our center (TOF-OSEM-PSF 3i/16s and a 5-mm gaussian filter) was used as the reference.

**Table 1:** Image reconstruction algorithms and the parameters used in the study

Reconstruction method	TOF	PSF	Iterations	Subsets	Gaussian filter (mm)	β-value
OSEM	No	No	3	16	5	n.a
TOF-OSEM	Yes	No	1,2,3,4,5,6	16	5	n.a
TOF-OSEM-PSF	Yes	Yes	1,2,3,4,5,6	16	5	n.a
TOF-OSEM-PSF	Yes	Yes	3	16	3, 8	n.a
BSREM	Yes	Yes	n.a	n.a	n.a	100, 200, 300, 400, 600, 800, 1000

*TOF*, time of flight; *PSF*, point-spread function; *OSEM*, ordered subsets expectation maximization; *BSREM*, block-sequential regularized expectation maximization

### Data analysis

All reconstructed scans (n = 440) were analyzed in the aQuant software (MedTrace, Hørsholm, Denmark).^18^ In short, aQuant uses a basis function implementation of the single-tissue compartment model, including spill-over parameters for left- and right-ventricular blood, to construct parametric images of the PTF.^19–22^ Blood time-activity curves were determined automatically using a cluster analysis approach.^19^ The PTF images were then used for automatic myocardial wall delineation and segmentation, and MBF and PTF were calculated for the coronary territories (LAD, RCA, LCX) using nonlinear least-square fitting of the operational equation of the single-tissue compartment model. Transmural MBF (MBFt) was calculated as the product of MBF and PTF.

All scans reconstructed with the clinical reference algorithm were analyzed fully automatically in aQuant with no user intervention. Then, two different analyses were performed:

1. Myocardial VOIs and arterial and venous VOIs were copied to all other reconstructed scans, which were then processed in aQuant, omitting the myocardial wall delineation and segmentation steps of the software. Hence, identical myocardial, arterial, and venous VOIs were used for all reconstructed images.
2. All reconstructed scans were analyzed in aQuant using automatic segmentation without user intervention.

Analysis 1 constitutes the pure theoretical impact from reconstruction methods on kinetic modeling and MBF calculations from ^15^O-water without being affected by different VOI definitions. Analysis 2 corresponds more to the clinical reality where each scan would be analyzed separately.

The effect of reconstruction method on clinical diagnosis was assessed using the algorithm defined by Danad et al.,^4^ where a patient was diagnosed as positive for ischemic heart disease when at least two adjacent segments had an MBF value below 2.3 mL⋅g^−1^⋅min^−1^.

### Statistics

To analyze average differences between all reconstructions and the reference, the non-parametric paired Wilcoxon signed-rank test was used. A two-sided *P* value < .05 was considered significant. Spearman rank correlation (ρ) was calculated using linear regression. Statistical analysis was performed in MATLAB and GraphPad Prism.

## Results

**Figure 1:**
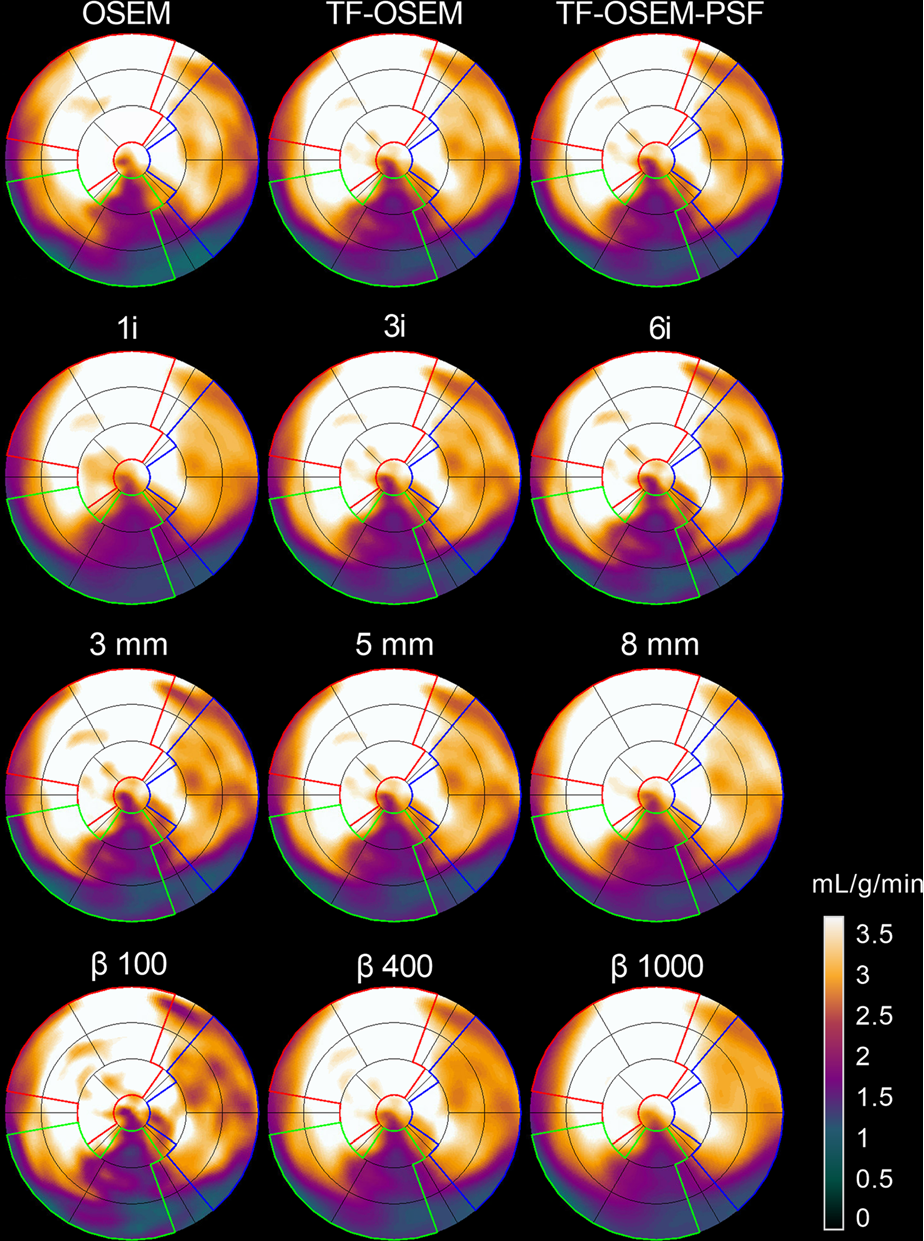
MBF polar plots showing minor visual differences between different reconstruction methods (fully automated analysis according to analysis method 2). On the top row are OSEM reconstructions with 3 iterations and a 5-mm filter, with and without TOF and PSF. The second row shows differences when varying the number of iterations, all three reconstructions are OSEM with TOF, PSF, and a 5-mm filter. The third row shows differences when varying the filter size, all three reconstructions are OSEM with 3 iterations, TOF, and PSF. On the bottom row are BSREM reconstructions with three different β-values

**Figure 2:**
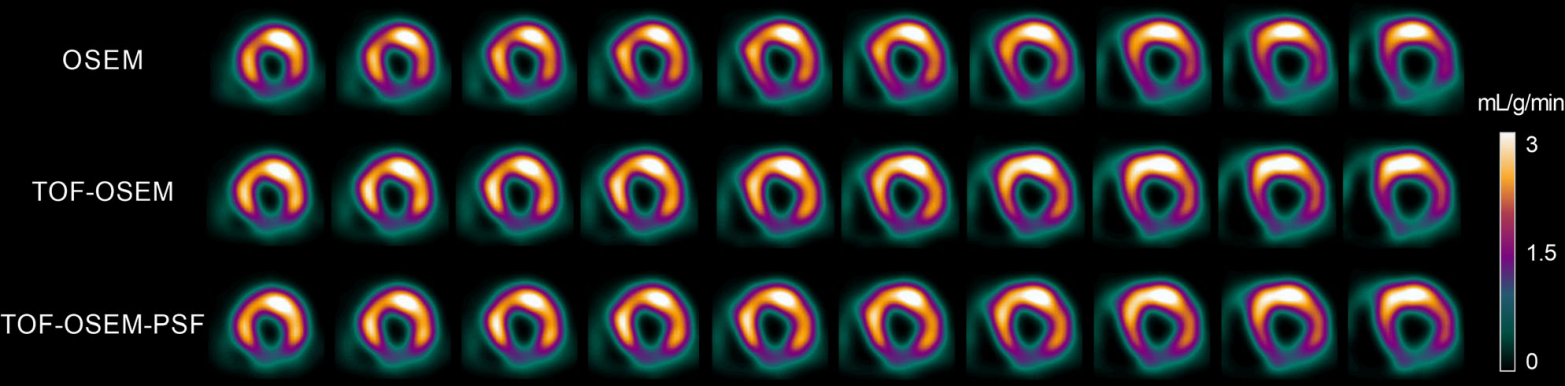
Short-axis MBFt images for three reconstruction methods using OSEM with 3 iterations and a 5-mm filter (fully automated according to analysis method 2). The top row is OSEM without TOF and PSF, the middle row is OSEM with TOF, and the bottom row is OSEM with TOF and PSF, which is the clinical standard method

Global hyperemic MBF values ranged between 1.1 and 3.9 mL⋅g^−1^⋅min^−1^ for all patients. One patient was excluded from the results due to obvious motion artifacts, giving a total of n = 418 reconstructed scans. In Figures 1 and 2, polar plots and short-axis parametric images from analysis 2 of MBF for different reconstruction settings are shown. In general, only subtle visual differences can be seen among the different reconstruction settings.

**Figure 3:**
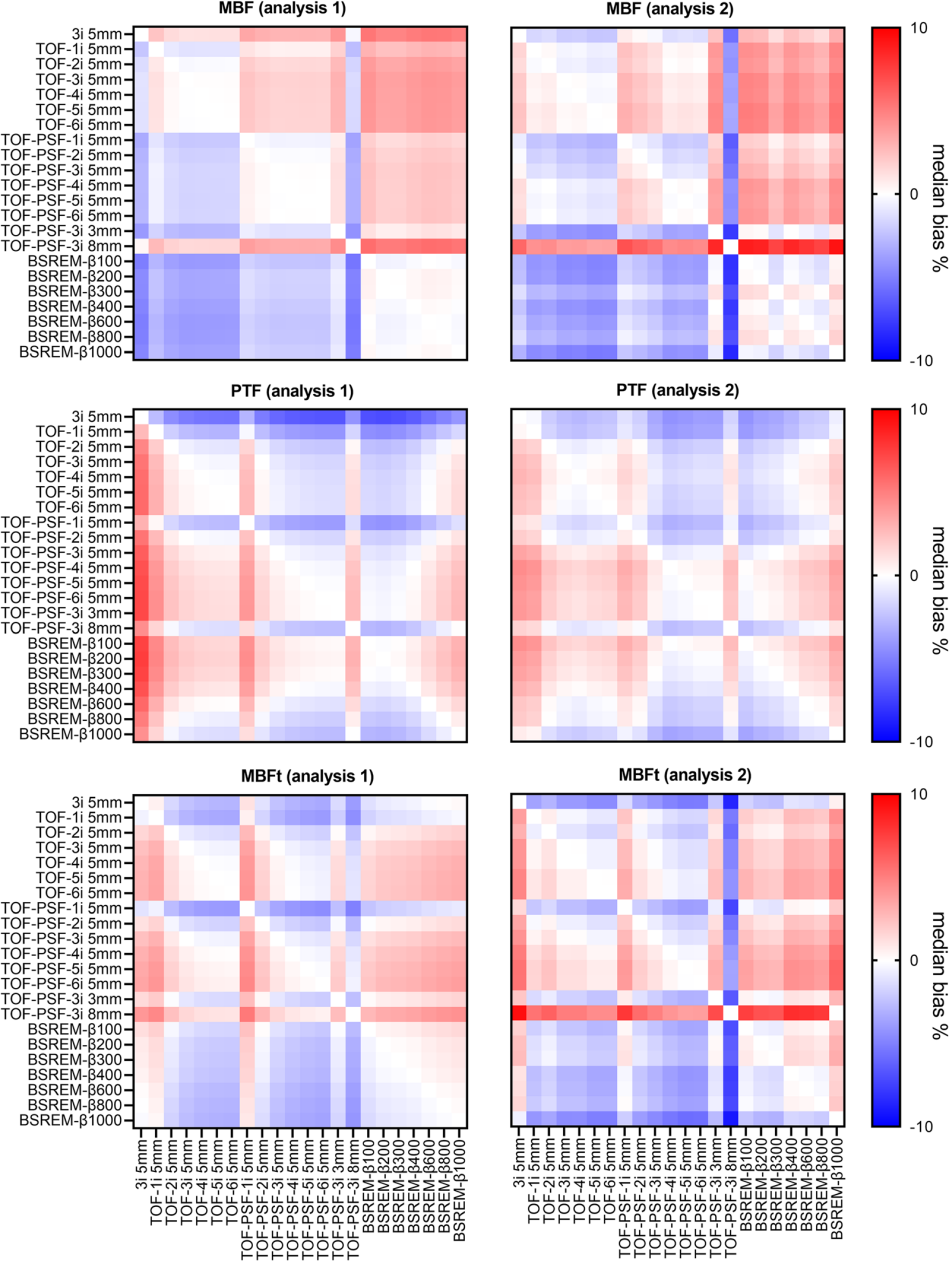
Heat maps showing median bias (%) of MBF (top), PTF (middle), and MBFt (bottom) for both analysis versions: copying myocardial, arterial, and venous VOIs from the reference reconstruction (analysis 1), and analysis by automatic segmentation for all reconstructions (analysis 2), left and right column, respectively

Across the two analyses and on the regional level, correlations to the reference reconstruction were strong for all parameters with ρ ≥ 0.95 for MBF, ρ ≥ 0.83 for PTF and ρ ≥ 0.91 for MBFt. Heat maps showing median biases among all reconstructions on the global level for MBF, PTF, and MBFt are shown in Figure 3. For all comparisons of MBF, PTF, and MBFt, biases were minor, although often statistically significant since effects of changes in reconstruction algorithms generally led to changes in the same direction for all patients.

Median bias (%) among all reconstructions compared to the clinical standard reconstruction was − 1.3 to 2 for MBF, − 3 to 1.5 for PTF, and − 2.7 to 1 for MBFt with analysis 1, and − 2.2 to 2.3 for MBF, − 3.5 to 0.6 for PTF, and − 4.5 to 2.2 for MBFt with analysis 2. The inter quartile range of the bias (%) was 0.1-5.3 for MBF, 0.2-2.6 for PTF, and 0.2-3.8 for MBFt with analysis 1, and 0.8-7 for MBF, 0.8-3.3 for PTF, and 1.3-5.3 for MBFt with analysis 2.

**Figure 4:**
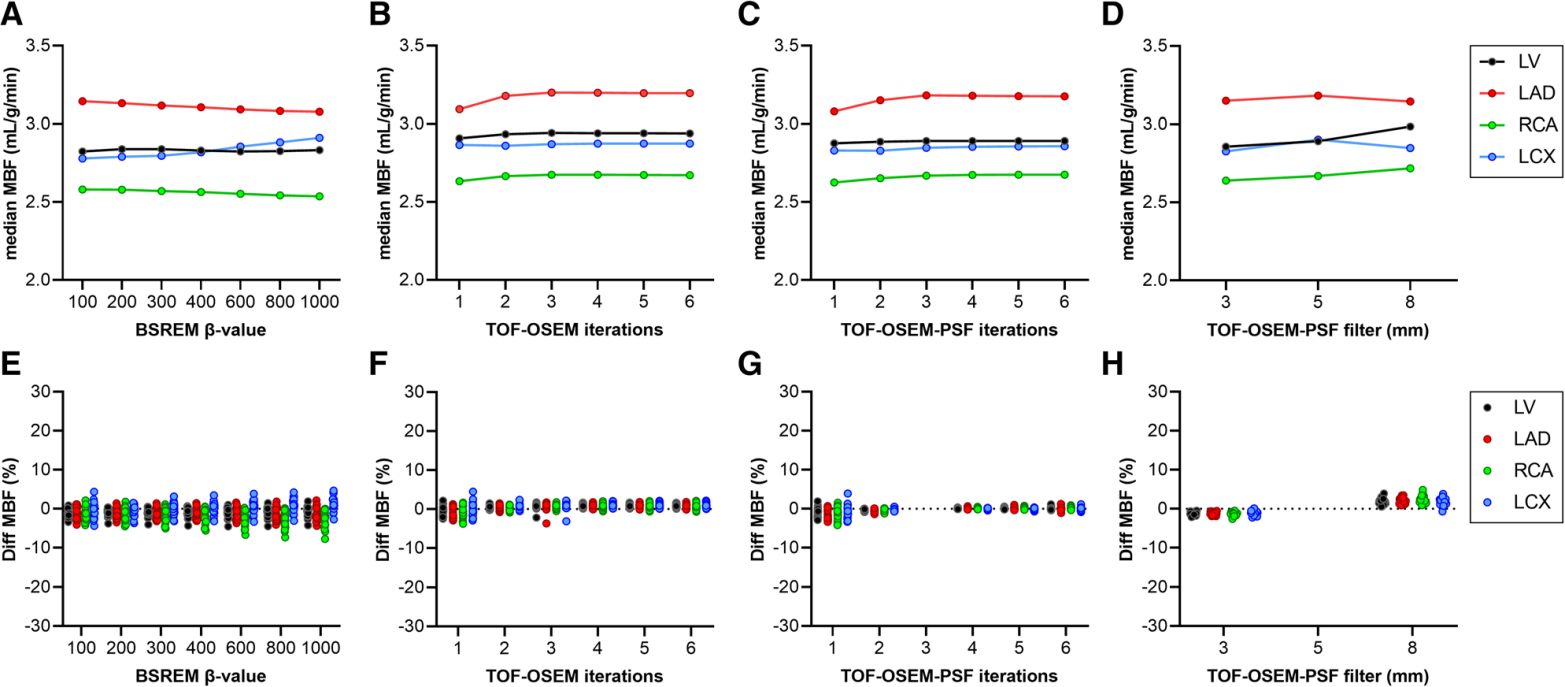
Impact on MBF quantification from changing β-value (**A**, **E**), number of iterations with TOF-OSEM (**B**, **F**), number of iterations with TOF-OSEM-PSF (**C**, **G**), and filter size (**D**, **H**). The top row shows the median value of all patients per data point and the bottom row are scatter dot plots showing the relative difference from the reference reconstruction (TOF-OSEM-PSF with 3 iterations, 16 subsets and a 5-mm filter) for each patient. Data are shown for the whole left ventricle (LV) and the coronary territories (LAD, RCA, and LCX). In this figure, myocardial VOIs and arterial and venous VOIs from the reference reconstruction were copied to all other reconstructed scans (analysis 1)

**Figure 5:**
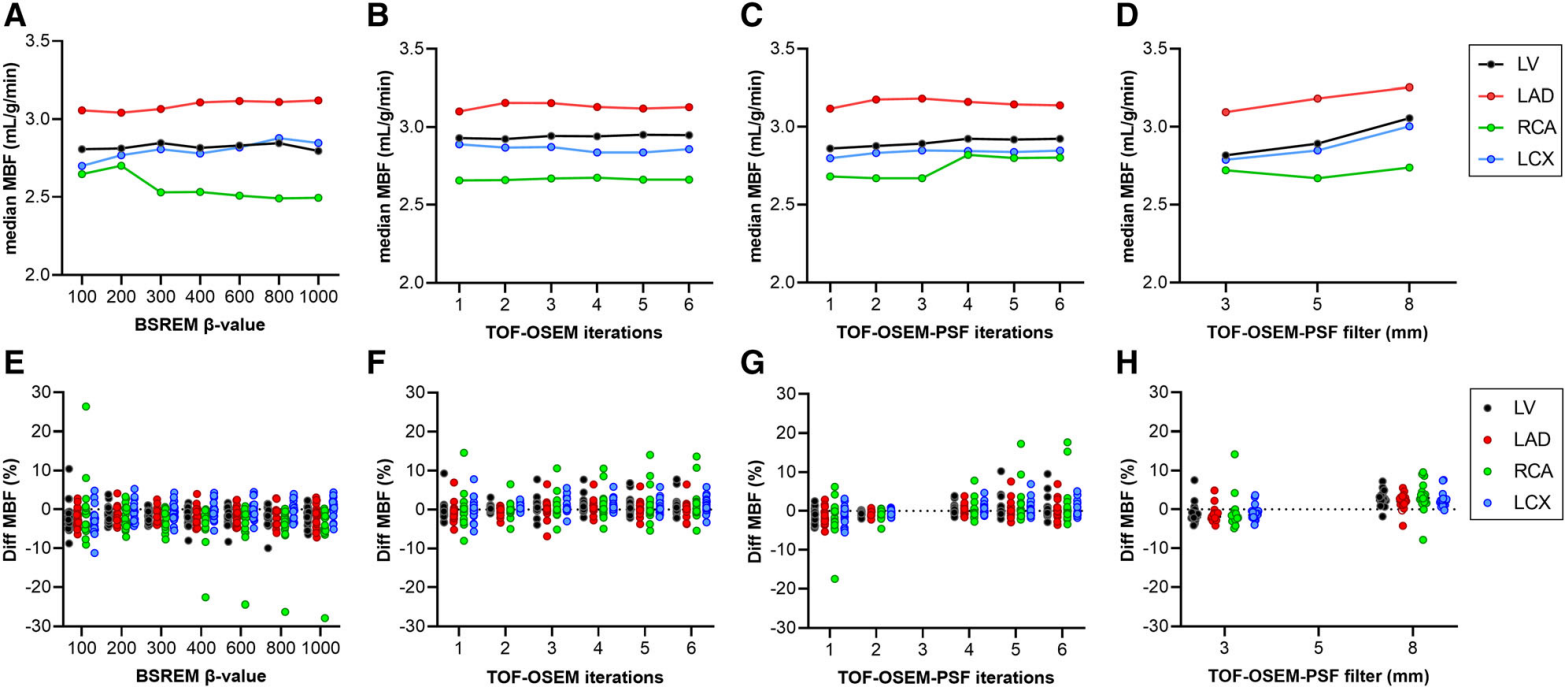
Impact on MBF quantification from changing β-value (**A**, **E**), number of iterations with TOF-OSEM (**B**, **F**), number of iterations with TOF-OSEM-PSF (**C**, **G**), and filter size (**D**, **H**). The top row shows the median value of all patients per data point and the bottom row are scatter dot plots showing the relative difference from the reference reconstruction (TOF-OSEM-PSF with 3 iterations, 16 subsets and a 5-mm filter) for each patient. Data are shown for whole left ventricle (LV) and the coronary territories (LAD, RCA, and LCX). In this figure, a fully automated analysis was used for all reconstructions (analysis 2)

In Figures 4 and 5, impact on MBF quantification is shown separately for different β-values, number of iterations with and without PSF, and filter sizes for both analyses, respectively. A minor trend of increased MBF in LCX with increased β-value with all analyses was seen in contrast to decreased MBF in RCA for analysis 2. Increasing the number of iterations did not impact the MBF, except for a slight increase in RCA with TOF-OSEM-PSF with 4, 5, and 6 iterations in analysis 2. A narrower filter than the standard 5 mm resulted in reduced MBF while a larger filter resulted in increased MBF with both analysis 1 and 2.

**Figure 6:**
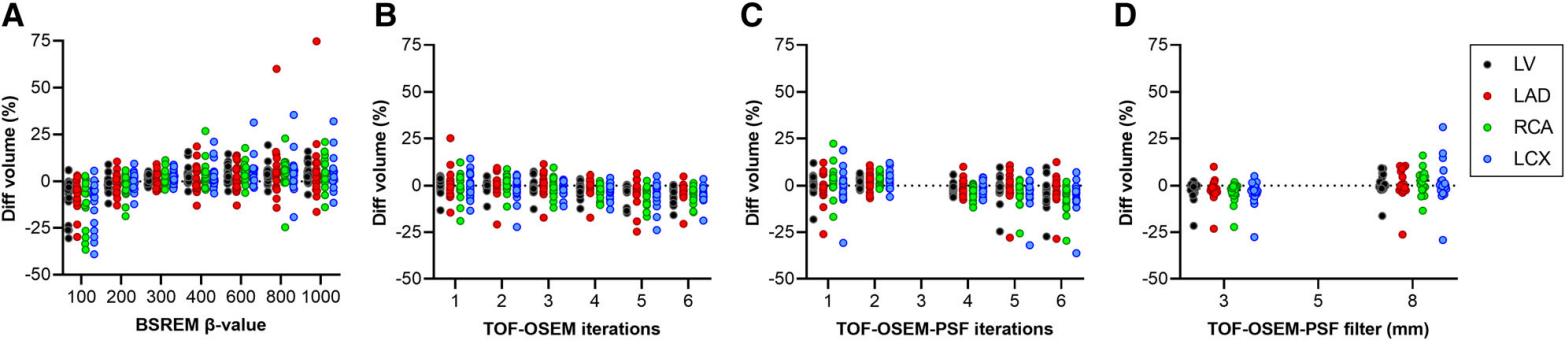
Scatter dot plots showing the relative difference in volume from the reference reconstruction (TOF-OSEM-PSF with 3 iterations, 16 subsets and a 5-mm filter) for each patient when changing β-value (**A**), number of iterations with TOF-OSEM (**B**), number of iterations with TOF-OSEM-PSF (**C**), and filter size (**D**). Data are shown for whole left ventricle (LV) and the coronary territories (LAD, RCA, and LCX). The data showcased in this figure were generated by automatic segmentation for all reconstructions (analysis 2)

Figure 6 shows scatter dot plots from analysis 2, displaying the relative difference of myocardial VOI volumes for each reconstruction compared to the clinical reference. Significant differences were seen for many reconstructions, indicating an impact on the automatic segmentation routine.

Possible changes in diagnoses in the standard clinical analysis (analysis 2) were studied using the ischemic cutoff at 2.3 mL⋅g^−1^⋅min^−1^. Using the clinical reference reconstruction, ten patients were diagnosed as ischemic (7 LAD, 10 RCA, 5 LCX). Compared to the clinical reference, one single patient changed diagnosis from positive to negative for four different reconstruction methods (BSREM β 100, OSEM without TOF or PSF, and TOF-OSEM and TOF-OSEM-PSF with 6 iterations).

## Discussion

In this study, the influence of a wide range of different image reconstruction settings on quantitative MBF, MBFt, and PTF measurements with ^15^O-water PET/CT was investigated. Data analysis was divided into two parts to study the impact on kinetic modeling in theory with copying of myocardial, arterial, and venous VOIs (analysis 1), and from a more clinical perspective with a complete automated analysis using the software that is clinically used at our institution (analysis 2). Overall, correlation between different reconstructions was high for all analysis methods, and biases in MBF, PTF, and MBFt values compared to those based on our clinical reference method was limited to a few percent, which is of the same order or less than the intra- and inter-observer variability as we have published previously.^17^

MBF from ^15^O-water is calculated using the clearance rate (*k*_2_) instead of the uptake rate (*K*_1_), which is used for all other perfusion tracers. One benefit of using *k*_2_ is its robustness toward PET-CT misalignment^18^ and several other previous studies have shown that MBF based on the clearance rate can be estimated accurately even without attenuation correction.^15,16,23,24^ One of these studies also showed only a minor impact on MBF from a smaller set of different reconstruction settings using ^15^O-water PET/MR data.^15^ This is in line with the results of the current study where clearance-based MBF is only minimally affected throughout both analyses, despite the use of extreme reconstruction settings, such as BSREM with a β-value of 1000 or TOF-OSEM with only one iteration or using three iterations with a large 8-mm filter.

Interestingly, impact on PTF and MBFt were also minor regarding both analysis methods. A stable PTF, regarding different reconstructions, is important because it holds diagnostic information in addition to MBF. PTF and especially the perfusable tissue index, which is defined as the ratio of PTF to the anatomical tissue fraction, has shown good correlation with scar burden measured by late gadolinium enhancement on cardiac MRI^25,26^ and with tissue data in a pig model of myocardial infarction.^27^

From Figures 4 and 5 it is notable that different number of iterations, filter sizes, β-values, and inclusion of PSF do have some effect on MBF values, and some trends can be observed. However, the changes are in the order of a few percent, and hence with limited clinical implications. Nevertheless, an increase in MBF with increased filter size can clearly be seen for both analysis 1 and 2, and an increase in MBF with increasing the number of iterations with the inclusion of PSF can clearly be seen for analysis 2. In the clinical, fully automated analysis 2, MBF in RCA decreases with increasing β-values, in contrast with the trend in the other regions. A reason for this is that with increasing β-values the automatic segmentation of the aQuant software extended the RCA VOI volume in the inferior direction, which led to an inclusion of partly abdominal regions in the RCA and, hence, a decreased MBF. This explains the four points in Figure 5E with large underestimations of MBF for β-values between 400 and 1000. These four points are all from the same patient where the software severely extended the RCA VOI volume in inferior direction. It should, however, be emphasized that such a severe extension of the inferior wall would have been detected by the observer and manually adjusted in clinical reality. It is further on evident from Figure 6 that myocardial VOI volumes were affected to a larger degree than MBF. The aQuant software utilizes radial profiles on parametric PTF images with a defined relative cutoff for the endo- and epicardium. Reconstructions with more smoothing will then lead to larger VOIs. Interestingly, this led to larger changes in MBF and MBFt than in PTF itself when the myocardial VOI time-activity curves were subsequently analyzed using nonlinear regression.

One limitation of this study is that the effect of number of iterations, TOF, and PSF is different depending on patient BMI, and whether the arms are up/down, etc., which we have not considered in this study as we used a random cross-section of patients referred for cardiac PET. We did, however, include patients with a large range of different BMIs. Furthermore, we could have used an even wider range of reconstruction methods, but the included algorithms essentially cover more than what would be considered clinically relevant.

In the clinical evaluation of ^15^O-water PET, a diagnostic cutoff at 2.3 mL⋅g^−1^⋅min^−1^ is used.^5^ The diagnosis in one patient was changed from positive to negative for ischemia for three of the most extreme variations of tested reconstruction methods. This patient had MBF values close to the diagnostic cutoff in the RCA region, where the diagnosis is affected by the inherent uncertainty of MBF quantification anyway. The diagnosis did not change for any other patient or reconstruction, indicating that the choice of reconstruction algorithm in clinical practice is probably not crucial for ^15^O-water PET, although a larger number of patients would have to be studied to draw firm conclusions. This also implies that it is likely that the type of PET scanner system would have limited effect on MBF, which is confirmed by a previous study where we showed a high agreement between MBF values measured in the same patients using a state-of-the-art digital PET/MR system and a previous generation BGO-based PET/CT system.^15^ The use of generally applicable hyperemic MBF cutoff values in clinical diagnosis requires robust quantification, and the low sensitivity of MBF values to reconstruction method and, as previously shown, scanner type and PET/CT misalignment, confirms that diagnostic cutoff values can be consistently used for ^15^O-water.

Clinical analysis 2 is a fully automated analysis using the aQuant software and is as such only valid for that software package. The impact from image reconstructions on other software packages may differ as they utilize different segmentation algorithms for the myocardial, arterial, and venous VOIs. However, as the results from the pure theoretical impact on the kinetic modeling of ^15^O-water (analysis 1) showed only minor differences across all reconstructions, it is likely that other software packages would yield a similar result as in this study with minimal impact of reconstruction method on MBF quantification from ^15^O-water.

## New Knowledge Gained

MBF from ^15^O-water PET is minimally affected by the choice of image reconstruction algorithm and the diagnostic cutoff can consistently be used across a wide range of reconstructions.

## Conclusion

Changes in reconstruction settings such as filter size, number of iterations, inclusion of time of flight or resolution recovery, and regularization, have minor impact on MBF values based on ^15^O-water PET analyzed using automated software. This study confirms that diagnostic MBF cutoff values can be consistently used for ^15^O-water. Impact on PTF was likewise minimal and can thus be used for viability measures across reconstructions.

## Supplementary Information

Below is the link to the electronic supplementary material.

Supplementary file1 (MP3 5676 kb)

Supplementary file2 (PPTX 1380 kb)

## Abbreviations

^15^O: Oxygen-15
PET: Positron emission tomography
CT: Computer tomography
BSREM: Block-sequential regularized expectation maximization
MBF: Myocardial blood flow
PTF: Perfusable tissue fraction
TOF: Time of flight
OSEM: Ordered subsets expectation maximization
PSF: Point spread function
MBFt: Transmural myocardial blood flow
