# Supplementary material for: Influence of image reconstruction on quantitative cardiac 15O-water positron emission tomography
Source: J Nucl Cardiol. 2022 Aug 4;30(2):716–25. doi: 10.1007/s12350-022-03075-5 (PMC10126040; doi:10.1007/s12350-022-03075-5)
Supplement: Supplementary file 2 — Supplementary file2 (PPTX 1380 kb) [file 12350_2022_3075_MOESM2_ESM.pptx]

## Slide 1
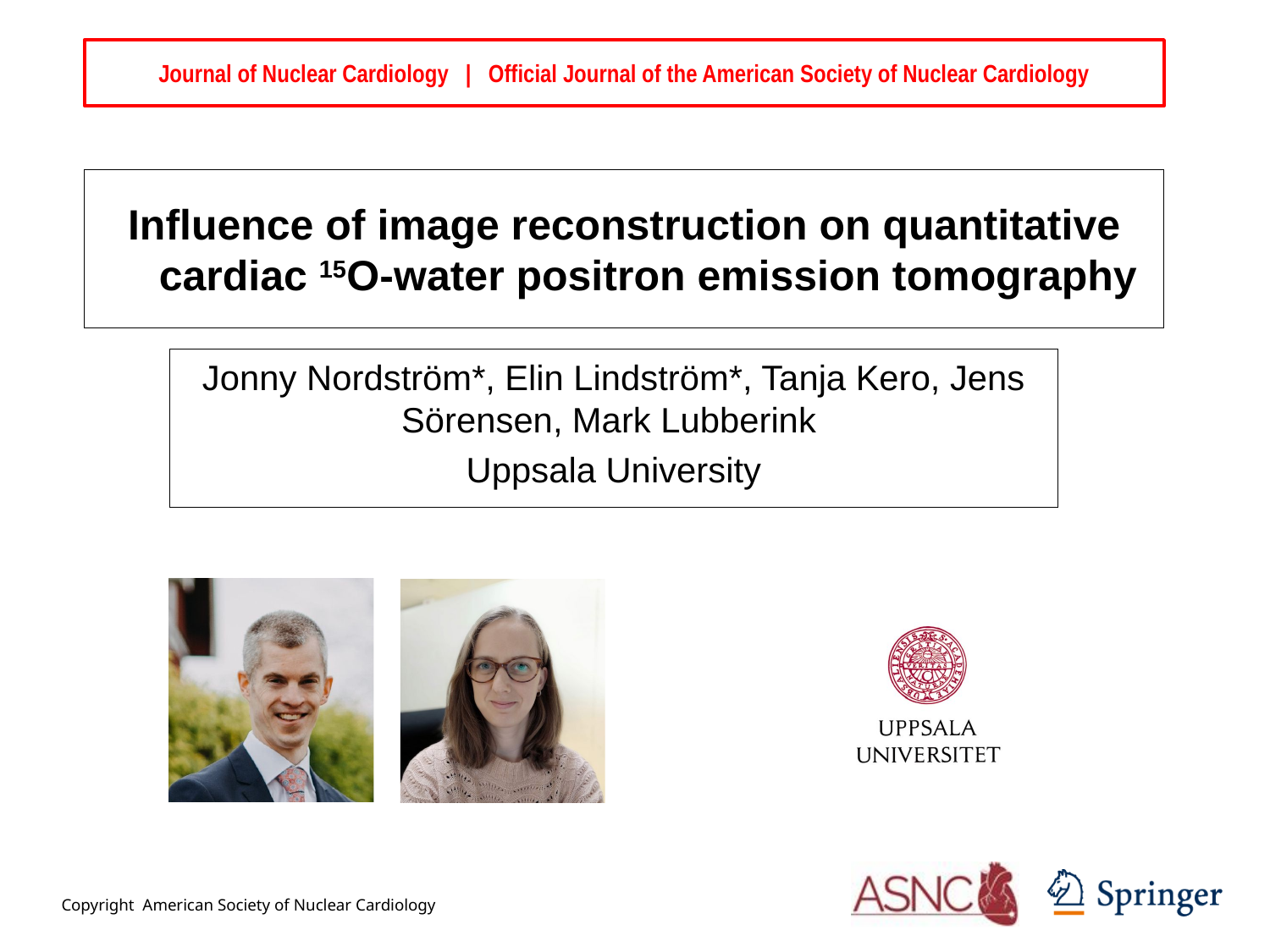

Journal of Nuclear Cardiology | Official Journal of the American Society of Nuclear Cardiology
# Influence of image reconstruction on quantitative cardiac 15O-water positron emission tomography
Jonny Nordström*, Elin Lindström*, Tanja Kero, Jens Sörensen, Mark Lubberink
Uppsala University
Copyright American Society of Nuclear Cardiology

## Slide 2
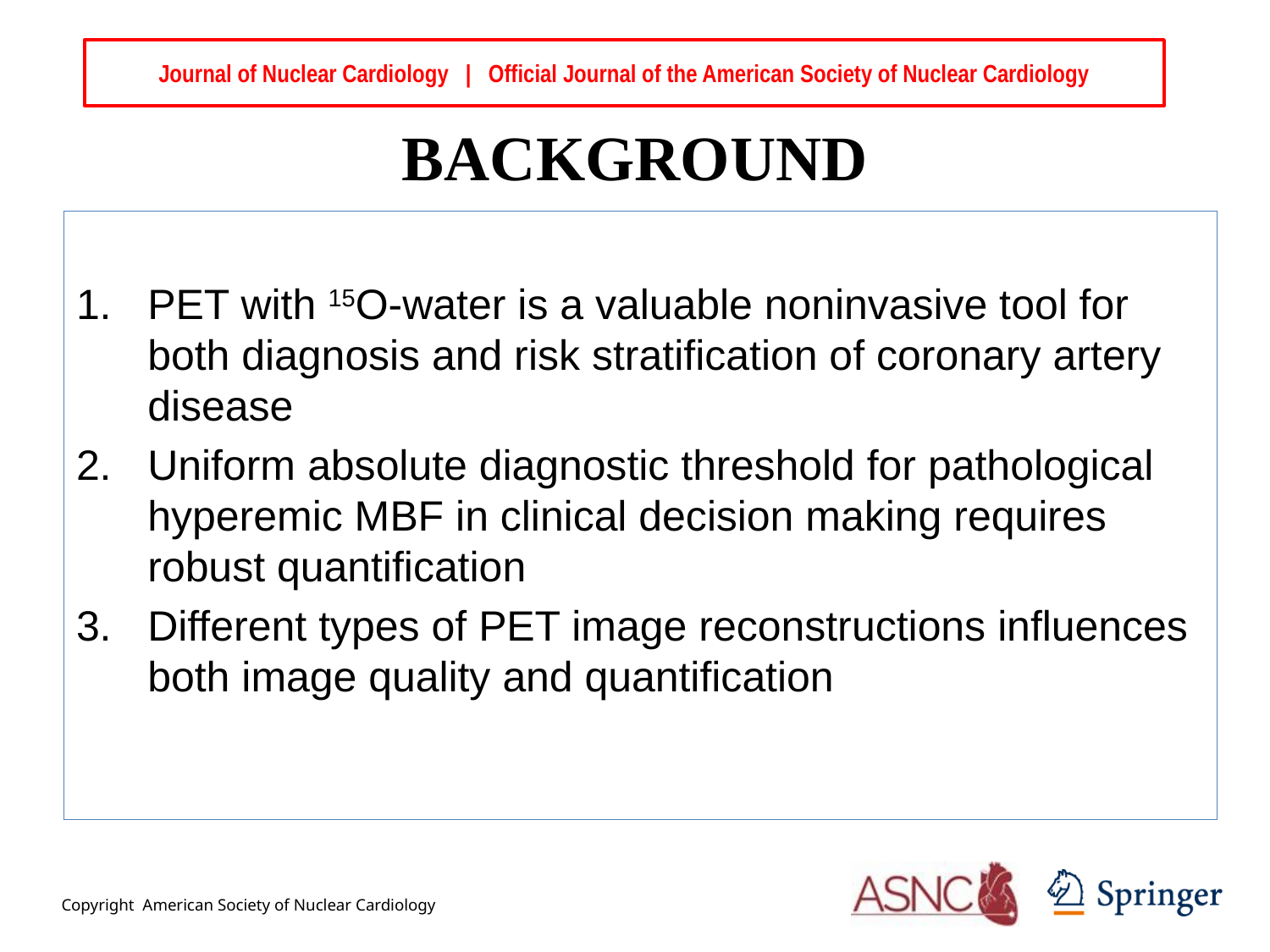

Journal of Nuclear Cardiology | Official Journal of the American Society of Nuclear Cardiology
# BACKGROUND
PET with 15O-water is a valuable noninvasive tool for both diagnosis and risk stratification of coronary artery disease
Uniform absolute diagnostic threshold for pathological hyperemic MBF in clinical decision making requires robust quantification
Different types of PET image reconstructions influences both image quality and quantification
Copyright American Society of Nuclear Cardiology

## Slide 3
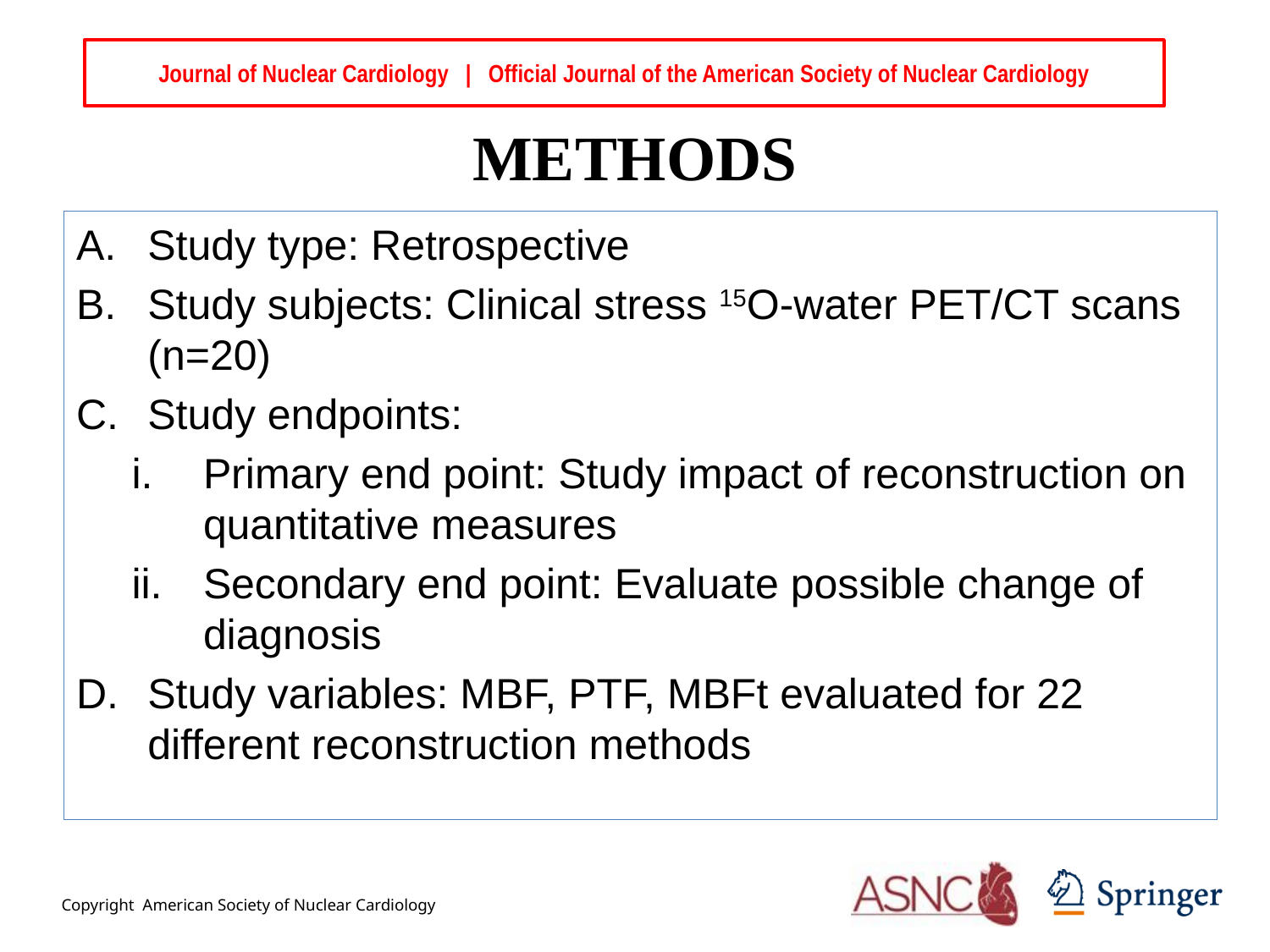

Journal of Nuclear Cardiology | Official Journal of the American Society of Nuclear Cardiology
# METHODS
Study type: Retrospective
Study subjects: Clinical stress 15O-water PET/CT scans (n=20)
Study endpoints:
Primary end point: Study impact of reconstruction on quantitative measures
Secondary end point: Evaluate possible change of diagnosis
Study variables: MBF, PTF, MBFt evaluated for 22 different reconstruction methods
Copyright American Society of Nuclear Cardiology

## Slide 4
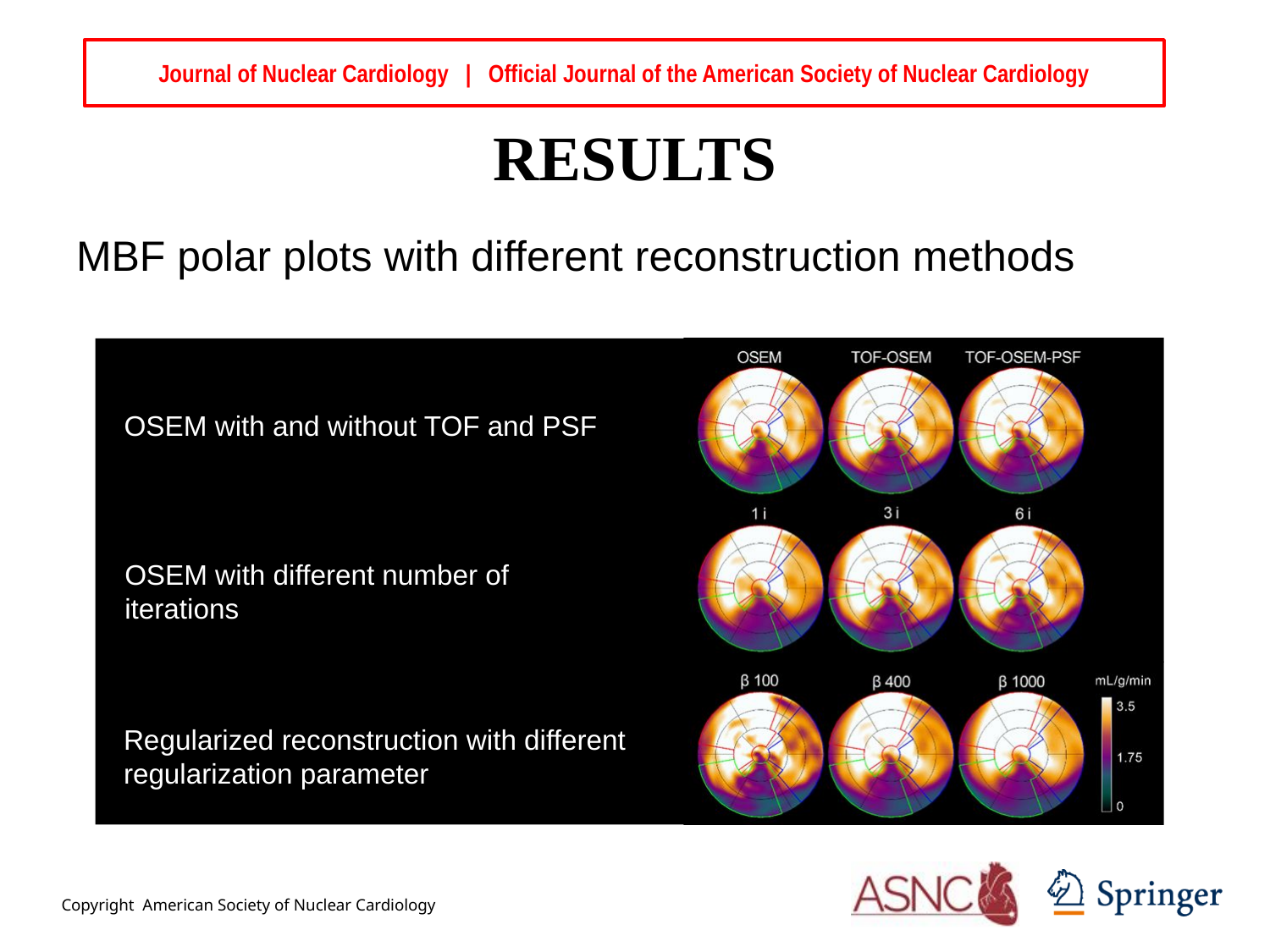

Journal of Nuclear Cardiology | Official Journal of the American Society of Nuclear Cardiology
# RESULTS
MBF polar plots with different reconstruction methods
OSEM with and without TOF and PSF
OSEM with different number of iterations
Regularized reconstruction with different regularization parameter
Copyright American Society of Nuclear Cardiology

## Slide 5
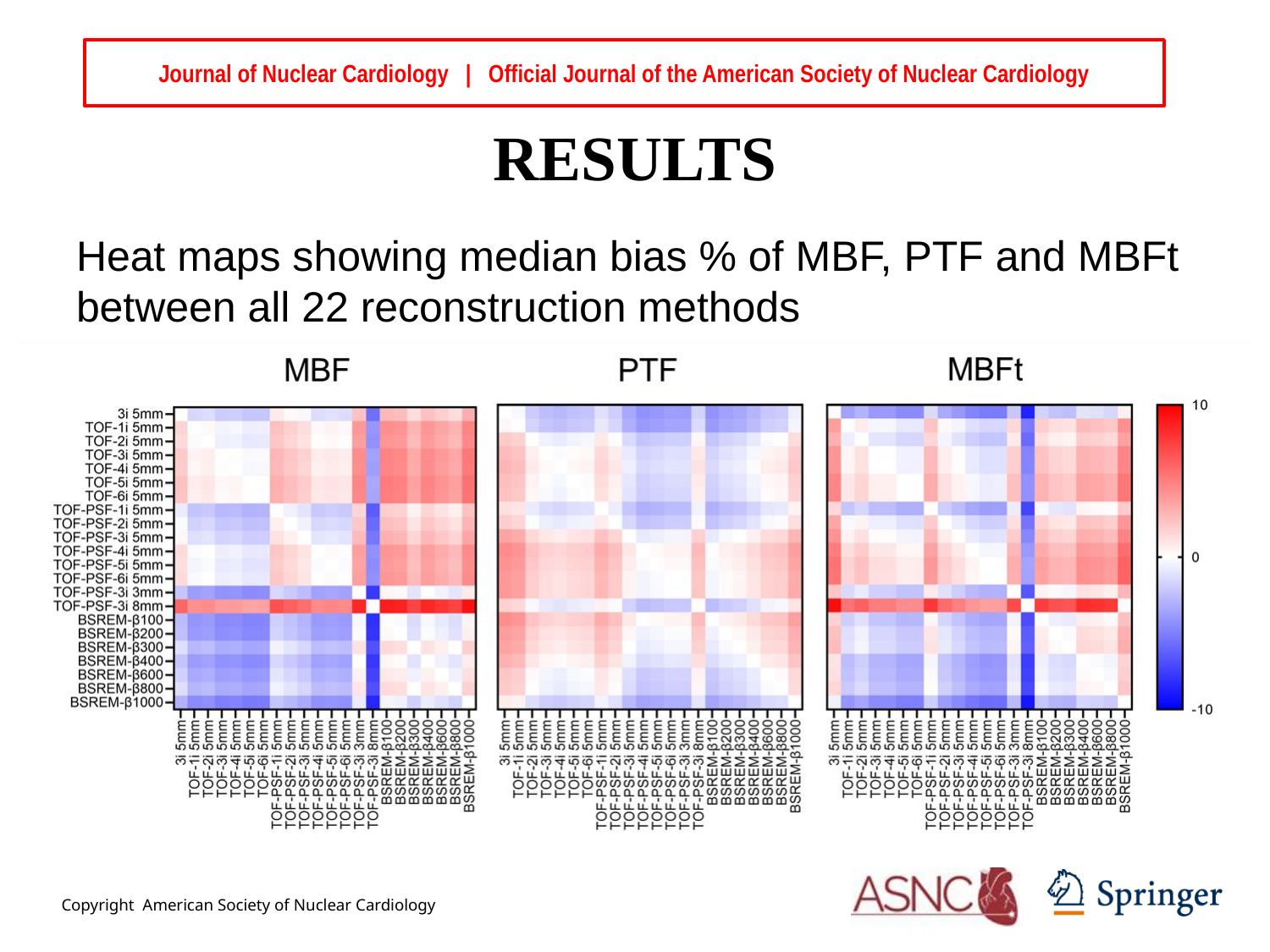

Journal of Nuclear Cardiology | Official Journal of the American Society of Nuclear Cardiology
# RESULTS
Heat maps showing median bias % of MBF, PTF and MBFt between all 22 reconstruction methods
Copyright American Society of Nuclear Cardiology

## Slide 6
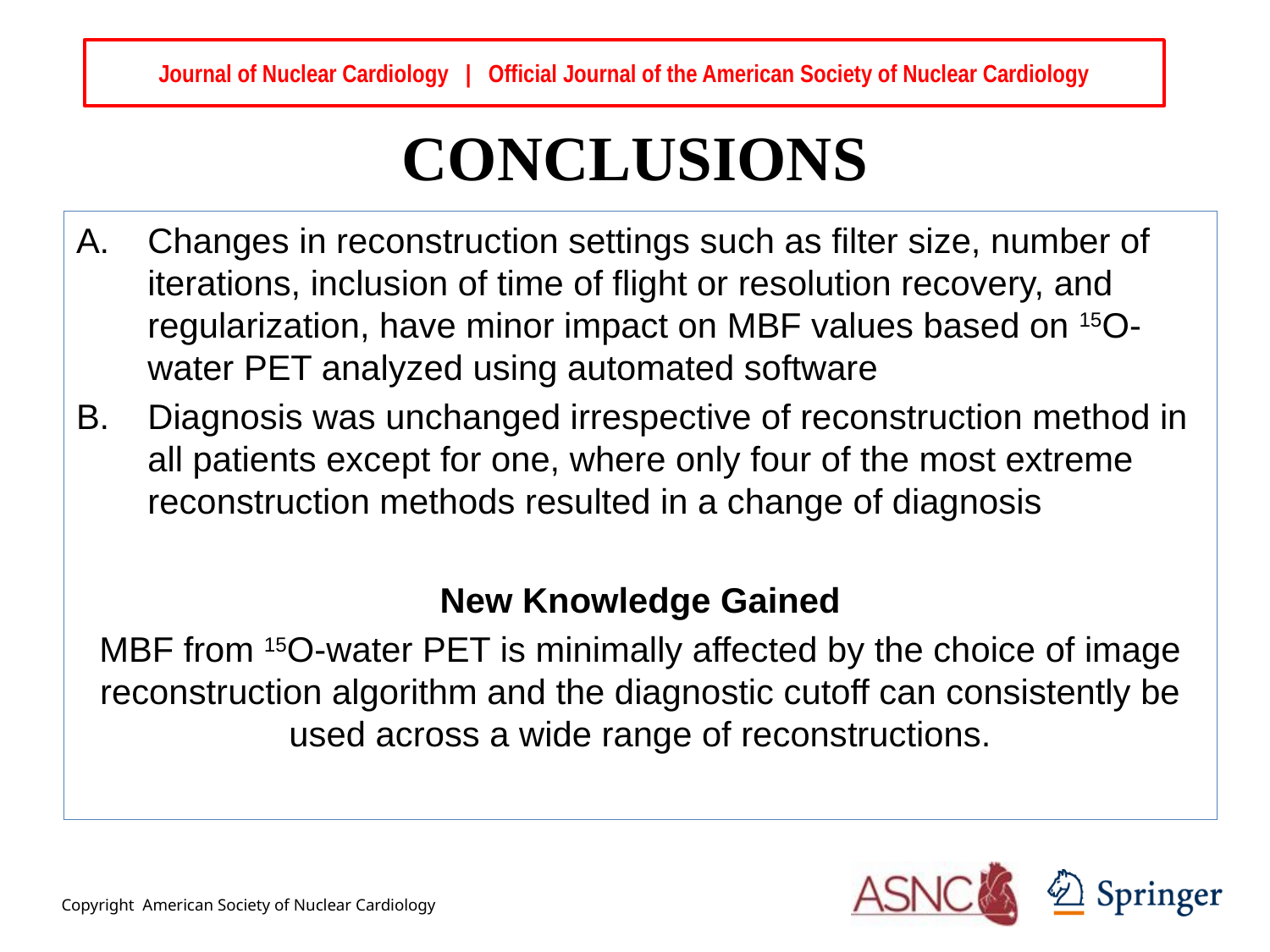

Journal of Nuclear Cardiology | Official Journal of the American Society of Nuclear Cardiology
# CONCLUSIONS
Changes in reconstruction settings such as filter size, number of iterations, inclusion of time of flight or resolution recovery, and regularization, have minor impact on MBF values based on 15O-water PET analyzed using automated software
Diagnosis was unchanged irrespective of reconstruction method in all patients except for one, where only four of the most extreme reconstruction methods resulted in a change of diagnosis
New Knowledge Gained
MBF from 15O-water PET is minimally affected by the choice of image reconstruction algorithm and the diagnostic cutoff can consistently be used across a wide range of reconstructions.
Copyright American Society of Nuclear Cardiology
